# Looking to Score: The Dissociation of Goal Influence on Eye Movement and Meta-Attentional Allocation in a Complex Dynamic Natural Scene

**DOI:** 10.1371/journal.pone.0039060

**Published:** 2012-06-29

**Authors:** Shuichiro Taya, David Windridge, Magda Osman

**Affiliations:** 1 School of Biological and Chemical Science, Queen Mary College, University of London, London, United Kingdom; 2 Centre for Vision Speech and Signal Processing, University of Surrey, Guildford, United Kingdom; Northwestern University, United States of America

## Abstract

Several studies have reported that task instructions influence eye-movement behavior during static image observation. In contrast, during dynamic scene observation we show that while the specificity of the goal of a task influences observers’ beliefs about where they look, the goal does not in turn influence eye-movement patterns. In our study observers watched short video clips of a single tennis match and were asked to make subjective judgments about the allocation of visual attention to the items presented in the clip (e.g., ball, players, court lines, and umpire). However, before attending to the clips, observers were either told to simply watch clips (non-specific goal), or they were told to watch the clips with a view to judging which of the two tennis players was awarded the point (specific goal). The results of subjective reports suggest that observers believed that they allocated their attention more to goal-related items (e.g. court lines) if they performed the goal-specific task. However, we did not find the effect of goal specificity on major eye-movement parameters (i.e., saccadic amplitudes, inter-saccadic intervals, and gaze coherence). We conclude that the specificity of a task goal can alter observer’s beliefs about their attention allocation strategy, but such task-driven meta-attentional modulation does not necessarily correlate with eye-movement behavior.

## Introduction

Eye-movements are involved in virtually all human activities. Because of limited retinal resolution and limited processing resources, seeking out information in a dynamic visual scene to support ongoing cognitive and behavioral activities requires constant redirection of gaze and attention. Indeed, there is strong empirical support showing a correspondence between eye-movements and a variety of demanding cognitive tasks [1]–[3]. The key concern of the present study is to address the following question: Given the need to redirect attention to ongoing tasks in visual scenes, how do we prioritize visual information that may be of relevance to the task at hand given the wealth of potentially relevant information that can be attended to at any one time?

Thus far, there have been two distinct theories of human attentional allocation and eye-movement control, and they make different claims about how prioritization of visual information processing is achieved. This can either be through top-down, goal-driven attentional selection or else via bottom-up stimulus-driven attentional selection. In the latter case, in its most extreme formulation, this approach proposes that eye-movements are purely stimulus-driven [4]–[10]. Influential computational models built on this premise implement the idea that our eyes are automatically attracted toward the most visually salient regions in a scene [4]–[6]. This implies the existence of topographically-organized ‘saliency maps’ of the scene which assign salience values to low-level visual features (image intensity, edge orientation, color, and motion). The contrary extreme top-down account instead proposes that observer’s fixations are purely controlled by goal oriented, top-down mechanisms [11]–[14]. Support for the latter hypothesis comes from evidence that eye-movements are strongly influenced by cognitive factors, such as contextual meaning, the observer’s knowledge, and the demands of the task [2], [3], [11]–[22]. In particular, for complex natural scene viewing, the claim is that visual saliency has a limited role in the guidance of our gaze, and that our gaze is directed toward sites that are important for understanding the meaning of the scene in the context of the ongoing task [11]–[13].

Given these competing approaches to understanding attentional allocation and eye-movement control, the primary concern of the present study is to investigate the effects of goal specificity on gaze control during natural, complex, dynamic scene-observation. Some work has already shown that task instructions can modulate observers’ eye-movement behavior [15]–[22]. For example, observers were told to either 1) memorize a scene for a later memory test, 2) search for a target object, or 3) freely view the scene without a particular goal in mind (free viewing). Contrasting with the memorizing task and the free viewing instruction, gaze allocation was specifically directed towards target objects only during the target objet search, i.e. when instructed to locate a specific object in the scene. More generally this implies that the specificity of the goal influences the way in which observers focus their gaze in a scene. However, to date, evidence indicating the influence of goal-specificity on gaze control is limited to studies using static scenes as visual stimuli [15]–[21], which leaves open the question as to whether this generalizes to dynamic scenes.

One reason that may limit the extent to which findings from static scenes generalize to dynamic scenes is that motion and temporal changes are strong predictors of eye-movement behavior e.g. [8], [23], [24]. This implies that a bottom-up mechanism driven by motion signals may play a dominant role in controlling gaze in dynamic scenes, in which case we would not expect to find goal specificity influencing eye-movements in dynamic natural scenes. In addition, dynamic scenes also differ from static scenes in consequence of the potential for temporal changes of location of task-relevant information. For instance, eye-movements have been recorded while observers are engaged in natural visuomotor tasks such as driving, walking, sports, making tea or making sandwiches [25]–[32]. These natural dynamic environments reveal an important aspect of eye-movement control, namely that eye-movements are tightly linked in time to the onset of the task. In general, these studies show that eyes are directed towards points in the scene that are most important for the spatiotemporal demands of the ongoing motor task. One concern with these studies is, however, that the changes in location of contextually-relevant points in the visual scene are entirely under the control of the observers taking part in the visuomotor tasks. This raises the question of how we adapt our gaze behavior with respect to the ongoing task in situations in which the task-relevant location changes independently from observer’s intention.

To bring clarity to the issues raised here concerning the influence of goal specificity on the processing of sequential information acquisition in dynamic natural scene viewing, the present study sets out to manipulate the goal-specificity of visual tasks in the context of the specific dynamic environment of recorded sport video. Eye-movements were thus recorded during the observation of video clips of singles tennis matches. In contrast to the types of static images used in previous studies to examine eye movement behavior in complex scenes, the tennis clips used in the present study included strong motion signals (ball and players). In addition there are several points where the ball’s motion changes abruptly either from a player’s hit or a bounce on the court/net, both of which are meaningful in the game context. We examined how the specificity of the goal modulates gaze guidance in a dynamic scene where contextually meaningful sites which can rapidly change in location over time. Goal specificity was manipulated in the following manner. We firstly presented observers with instructions simply to observe the scene, which we hypothesized would encourage purely stimulus-based processing of the scene (Non specific goal - NSG). We secondly presented observers with instructions to observe the scene with the purpose of answering a specific question at the end of the observation, which we hypothesized would encourage task-based top-down processing (Specific goal - SG).

**Figure 1:**
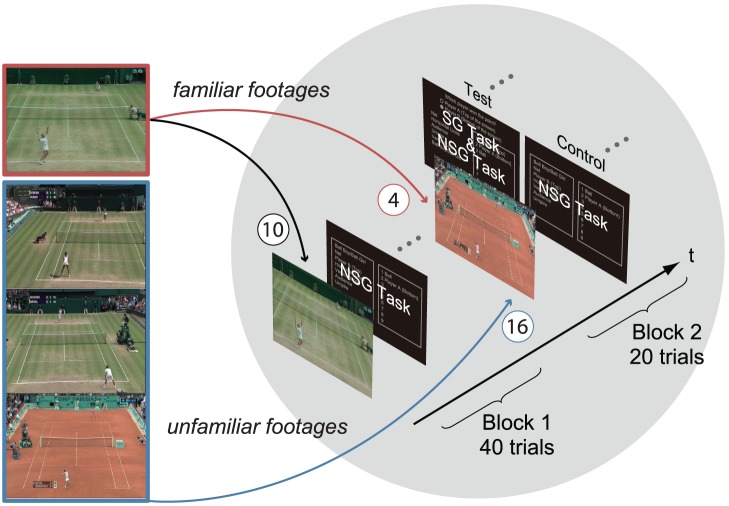
Stimuli and sequence of the current experiment.

The experiment was divided into two blocks (Figure 1). The first block (Block 1) consisted of 40 trials and was directly followed by the second block (Block 2) consisting of 20 trials. For both blocks, in every trial observers were presented with a short clip of a singles tennis match in which a point was played (25 Hz, 720×576 pixels/frame, mean ± SD = 9.3±1.7 s, with audio footage). In Block 1, 10 clips were presented four times in a blocked random order. In Block 2, 20 clips were presented once in random order. In Block 2, four out of 20 clips were selected from the clips presented in Block 1 and so were familiar to observers (*familiar footage*), and the remaining 16 clips were unfamiliar to observers (*unfamiliar footage*). In Block 1 as well as Block 2, after every clip presentation observers were asked to order items from the scene that they attended to (i.e. the NSG task). Observers were required to order the nine items; i.e. Ball, Player A (top of the display), Player B (bottom of the display), Net, Vertical court lines, Horizontal court lines, Ball boy/girl, Umpire, Audience, from the most attended to the least attended. These items were presented in a list box with an instruction that read, “From the list of options presented in the box, select them in order starting with the item you looked at most through to the item you looked at the least”. It was emphasized that “looked at” referred to the paying of attention to. In Block 2, along with the ranking task, observers were also required to indicate which of the two players had won the point (i.e. the SG task). Twenty observers took part in the experiment (test group). As a control, an additional 20 observers were also recruited and simply performed the NSG task both in Block 1 and Block 2 (control group). The primary goal of this study was to see whether the SG task could alter observers’ eye-movement behavior in dynamic scene observation. If goal specificity significantly modulates eye-movement behavior, then the between-block difference would be located only in the test group.

By using the NSG task (subjective ranking) we also consider a further issue concerning whether task specificity effects meta-attentional allocation. Recent studies have demonstrated that observers could not correctly monitor where they allocated their attention [33], [34]. For example Kawahara has shown that observers overestimate the area that they attended to in a static picture (e.g., a still image of traffic scene) [34]. However, the connection between observer’s belief about where they look and where they actually look is unclear. Therefore, we investigate the following question: Does the specificity of the goal of a task change meta-attentional loci, eye-movement pattern, or both?

## Results

### Effects of the Goal Specificity on Subjective Ranking

**Figure 2:**
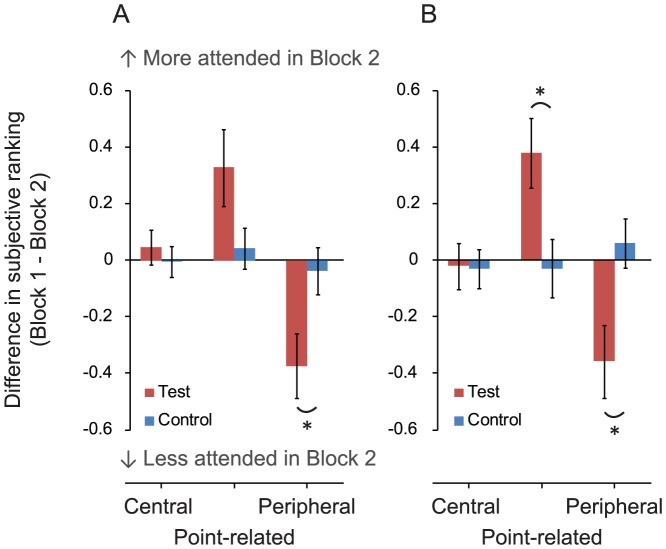
Results of the subjective ranking task. (A) The results of all pieces of footage. (B) The results of ‘familiar footage’. The plots show the between-block difference (i.e. Block 1 - Block 2) of the ranked order for each item category; i.e. ‘central items’ (Ball, Player A, Player B), ‘point-related items’ (Net, Vertical court lines, Horizontal court lines), and ‘peripheral items’ (Ball boy/girl, Umpire, Audience). Error bars are 1 standard error of the mean (*N* = 20). Note that in the ranking task the more an item attended, the smaller value (rank) would be assigned - thus if an item was attended more in Block 2 than Block 1, the difference (Block 1 - Block 2) would be positive, and vice versa.

Figure 2A shows the changes in subjective ranking between Block 1 and Block 2 (i.e. Block 1 - Block 2). Here the nine items were grouped into ‘central items’ (Ball, Player A, Player B), ‘point-related items’ (Net, Vertical court lines, Horizontal court lines), and ‘peripheral items’ (Ball boy/girl, Umpire, Audience) on the basis of the result of a cluster analysis (using Ward’s method [35] with squared Euclidean distances). The cluster analysis was conducted on the 40 observers’ average rank for the nine items. The three clusters were defined by the lowest level of chunking of the items in a hierarchical dendrogram implemented in IBM PASW Statistics 18 statistical package. As seen in Figure 2A, the test group observers ranked the ‘point-related items’ higher and ranked the ‘peripheral items’ lower in Block 2 than in Block 1, while the control group showed no between-block difference in the ranking for any of the item categories. A Mann-Whitney U test revealed a significant difference between the two groups based on ‘peripheral items’ (*p* = .041), but no significant difference between groups in ranking judgments for ‘central items’ and ‘point-related items’ (*p*>.2). By calculating the changes in ranking for the four clips which were presented both in Block 1 and Block 2 (i.e. familiar footage, Figure 2B), the between-group difference is significant in the ‘point-related items’ (*p* = .021) and the ‘peripheral items’ (*p* = .013), but again non-significant in terms of group difference in the ‘central items’ (*p*>.6). These results suggest that, according to an immediate retrospective recalling, participants observing the game with SG believed that they allocated more attention to items relevant to making a point decision, and allocated less attention to items irrelevant to making that decision.

### Saccadic Amplitudes

In order to test the effect of SG on eye-movement behavior, we first examined saccadic eye-movements. Saccadic eye-movements are important for rapid and sequential information acquisition and therefore may play an important role in dynamic scene observation [27], [29], [30]. However, it is well established that our visual sensitivity is considerably impaired during saccades (i.e. saccadic suppression, [36]). This implies that our visual system needs to control saccades so that we can maximize the information gain by utilizing rapid saccadic eye-movements while minimizing information loss caused by the saccadic suppression. Taking into account this trade-off, we expected that if the observers are carrying out a specific goal task, in order to minimize information loss they will generally make smaller saccades. It is also plausible that observers make smaller saccades in order to impinge their gaze onto a target location more precisely. Indeed, recent studies have reported that saccadic amplitudes varied according to the task requirements during static scene observation [20] (but see also [18]).

**Figure 3:**
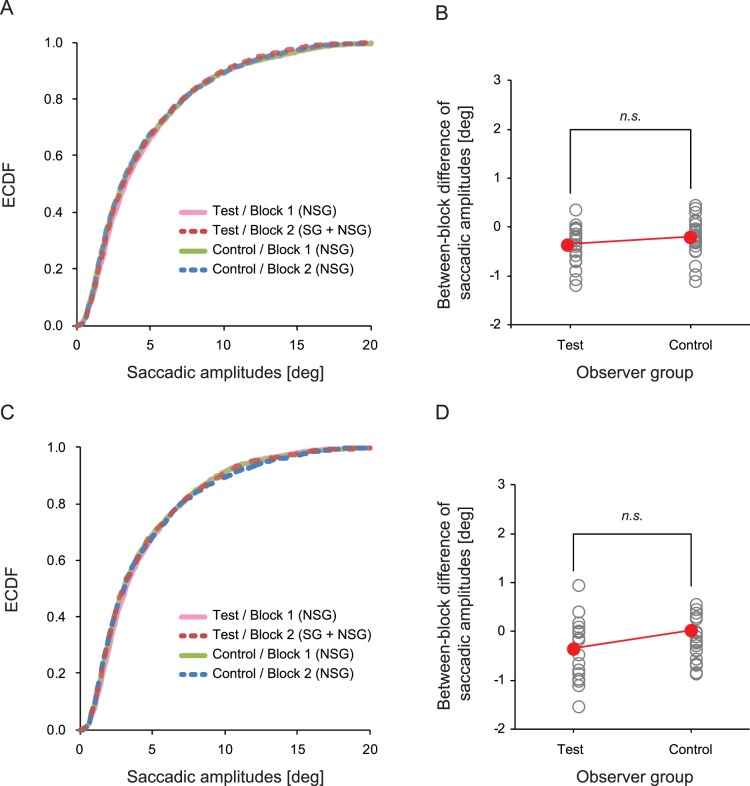
Distribution of saccadic amplitudes. (A) Empirical cumulative distribution functions (ECDFs) of saccadic amplitudes obtained with the eye-tracking data from all footage and (C) that obtained with the eye-tracking data from the ‘familiar footage’. (B) Group average (red circles) and individual data (open circles) of the between-block difference of saccadic amplitudes (Block 2 average - Block 1 average), calculated with the eye tracking data from all pieces of footage, and (D) that calculated with the eye-tracking data from the ‘familiar footage’.

Saccades were defined when eye velocity or acceleration exceeded a threshold, i.e. velocity >50 deg/s or acceleration >8000 deg/s^2^. The distribution of saccadic amplitudes is shown in Figure 3A. As shown in this figure, the distributions of saccadic amplitudes in the two groups almost overlap, suggesting no effect of SG on the saccadic amplitudes. To quantitatively assess the difference between the test group and the control group, we calculated the between-block difference of saccadic amplitudes (i.e. Block 2 - Block 1) for each group (Figure 3B). A Mann-Whitney U test on the calculated values between the two groups revealed no difference in the changes in saccadic amplitudes between the two groups (*p*>.3).

Only 20% of clips in Block 1 were also presented in Block 2 (familiar footage). This means that the contents of video clips (i.e. number of rallies, ball speed, and player’s movements etc) in the two blocks were very different. Thus simple comparison between two blocks might be affected by the difference in content. To avoid contamination by this effect, and in order to directly assess the effect of SG, we extracted data from familiar clips only (Figure 3C and 3D). Again, we found no difference in saccadic amplitudes between the two groups (*p*>.2, by Mann-Whitney U test).

### Inter-saccadic Intervals

Previous studies have indicated that measures of fixation durations can also be used as an index of the effect of ongoing tasks on eye movements e.g. [18], [20]. However, implementing a traditional fixation parsing algorithm for eye-tracking data with dynamic scene viewing is problematic. This is mainly because moving objects in a dynamic scene cause smooth pursuit eye-movements which can result in artificially elongated fixation durations [23], [24]. Instead of using fixation durations, we calculated inter-saccadic intervals (ISIs), i.e. the duration between one saccade-end to the next saccade-start. This measure would not only include fixations but also all other non-saccadic eye-movements (e.g. pursuit and optokinetic nystagmus). Thus, the measure would provide us a way of indexing how long eyes remained in a relatively small area (see below) between two consecutive saccades. Also, the measure allowed us to examine whether SG affected the duration of this eye movement behavior.

We calculated the ISIs from all of the saccades defined above. The mean of the eye-movement distance between one saccade-end and the next saccade-start was 0.71 deg (SD = 0.64) in visual angle for the test group observers and 0.74 deg (SD = 0.78) for the control group observers. About 85% of the inter-saccadic eye-movement distances were smaller than 1.2 deg (i.e. within the fovea) and 97% of them were less than 2.4 deg, thus eyes were kept in a small area between two consecutive saccades in most cases.

**Figure 4:**
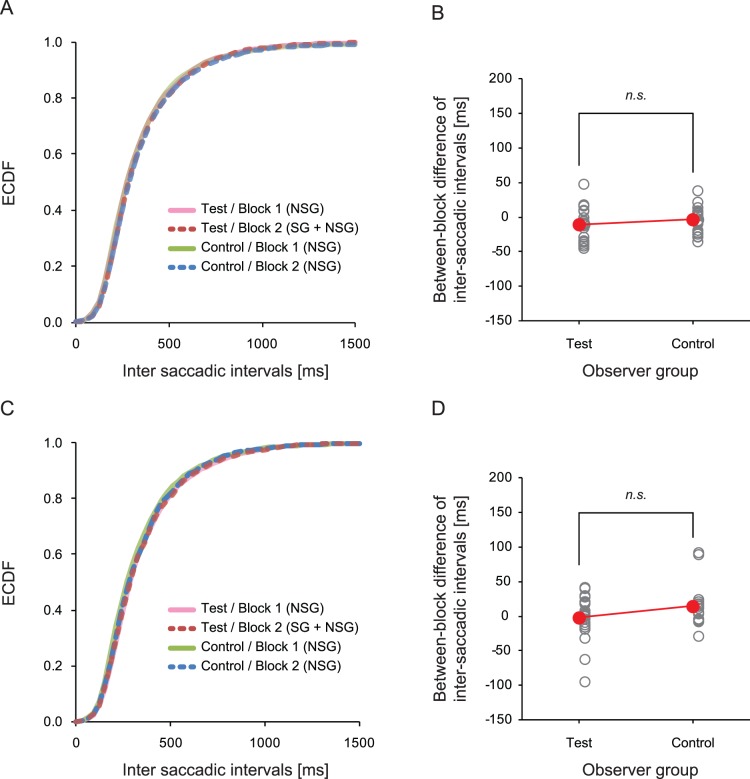
Distribution of inter-saccadic intervals (ISIs). (A) Empirical cumulative distribution functions (ECDFs) of ISIs obtained with the eye-tracking data from all footage and (C) that obtained with the eye-tracking data from the ‘familiar footage’. (B) Group average (red circles) and individual data (open circles) of the between-block difference of ISIs (Block 2 average - Block 1 average), calculated with the eye tracking data from all pieces of footage, and (D) that calculated with the eye-tracking data from the ‘familiar footage’.

The distribution of ISIs is shown in Figure 4A. The overlapped empirical calmative distribution functions (ECDFs) in the figure suggests that SG did not have an effect on this eye-movement measure. We also calculated the ISIs during observer’s exposure to the familiar clips only, and again found that they overlapped with ECDFs (Figure 4C). This means that the SG did not affect ISIs after having controlled for the content of the video clips. We conducted a Mann-Whitney U test on between-block difference of ISIs (Block 2 - Block 1) between the two groups, which also showed no statistical difference between the two groups either in the data from all clips (Figure 4B, *p*>.2) or just from familiar clips (Figure 4D, *p*>.2).

### Gaze Coherence

Next, we tested the effect of goal specificity on inter-observer coherence of the eye-movement pattern. This analysis was motivated by previous investigations that have conducted eye-movement recording during dynamic scene observation. Dorr et al. [24] showed that when watching a naturalistic movie (e.g. videos of everyday scenes and Hollywood action movie trailers), the inter-observer coherence of gaze location increased when isolated objects in the scene started to move. This suggests that motion is a strong bottom-up determiner of where we look. On the other hand, inter-observer gaze coherence was higher in Hollywood action movie trailers than non-professional edited videos of everyday scenes. This is probably because the location attracting observer’s interest in the non-professional videos was more dispersed than the professional videos. This result suggests that top-down factors such as motivational interest can modulate gaze similarity in dynamic scenes. We wanted to know whether the goal specificity could affect similarity of gaze pattern when watching short clips of a tennis game, which are likely to have a strong bottom-up motion signal (e.g. ball and players). We expect that the gaze of observers in the test group should be more attracted toward the goal-related contents, which should lead to an increase in eye-movement coherence. The gaze of observers in the control group should deviate because of individual differences in the interest points, which in turn should lead to lower inter-observer coherence of gaze location.

To calculate inter-observer gaze coherence we identified the ‘foveation’ locations [23], which include all non-saccadic eye-movements as an index of the center of gaze. The foveation location was identified using a method similar to that used in a previous study [23]. The 1000-Hz raw data were down-sampled into 25 Hz records of coordinates to obtain the gaze location for each frame. Meanwhile, the SR Research saccade parsing algorithm was used on the original 1000-Hz raw data to identify blinks and saccades. The frame-based samples were then labeled as foveation if the corresponding sample in the 1000-Hz raw data was not identified as either blinks or saccades.

We evaluated the inter-observer gaze coherence by adopting the method called Normalized Scanpath Saliency [10], [24], [37] on the foveation data. The NSS value for the test group and the control group was calculated independently. In essence, this method calculates gaze similarity based on the Gaussian-weighted distance between two gaze locations in a frame of footage. The NSS value for each frame was calculated in the following manner. First, we used the foveation data obtained from a single observer from one of the groups (we here refer to this observer the ‘reference observer’) in order to create a frame-based ‘foveation map’. In a foveation map, values were assigned for each one of a total of 720×576 pixels via the centering of a 2D Gaussian filter of 1.2 deg *δ* (size of fovea) on the foveation location. Second, the foveation map was normalized to a mean of 0 and a standard deviation of 1 to obtain a NSS map. Third, the foveation locations of the remaining 19 observers from the same group were mapped onto the obtained NSS map, and the sum of the values in the map for all of these locations was calculated. The same procedure was then repeated 20 times so that every observer was used as the reference observer. Finally, the mean of the 20 values obtained was calculated and used as the NSS value for a given frame. These procedures were repeated for all frames and all clips.

**Figure 5:**
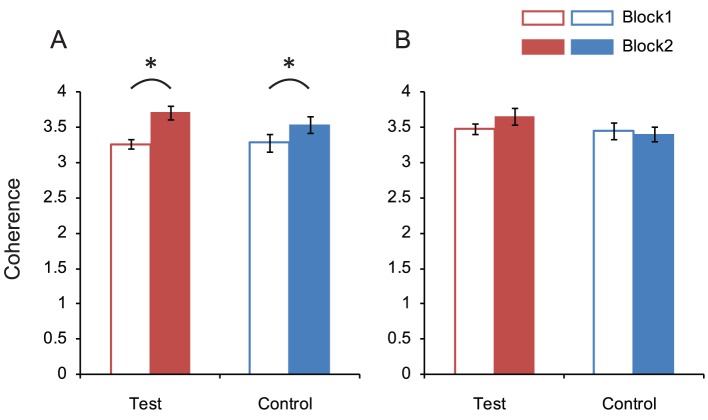
Inter-observers coherence of gaze location. (A) Normalized Scanpath Saliency (NSS) obtained with the eye-tracking data from all pieces of footage and (B) that obtained with the eye-tracking data from the ‘familiar footage’. Error bars indicate 1 standard error of the mean (*N* = 20).

Figure 5A plots the NSS averaged across all frames and all clips. In this figure, the NSS of the first 20 frames were omitted because a fixation point was presented every time before the start of footage, which strongly directed gaze to the center of the display in the earlier period of the video clip. Overall, it was the case, not only in the test group but also in the control group, that the average NSS value was larger in Block 2 than Block 1, despite the differences in task requirements between groups in Block 2: two-way mixed design ANOVA (group × block) revealed a significant main effect of block (*F*_1,38_ = 48.46, *p*<.001), but the main effect of group and interaction were non-significant (*F*_1,38_ = 0.33, *p* = .6; *F*_1,38_ = 3.72, *p* = .06, respectively). The larger NSS value in Block 2 may have been influenced by the difference in the video clip contents between the two blocks. To test this assumption, we calculated the average NSS value for clips appearing in both blocks (i.e. familiar footage) and indeed found no significant effects (Figure 5B): the main effect of block, main effect of group and interaction were non-significant (*F*_1,38_ = 1.24, *p* = .27; *F*_1,38_ = 1.06, *p* = .31; *F*_1,38_ = 3.66, *p* = .06, respectively).

**Figure 6:**
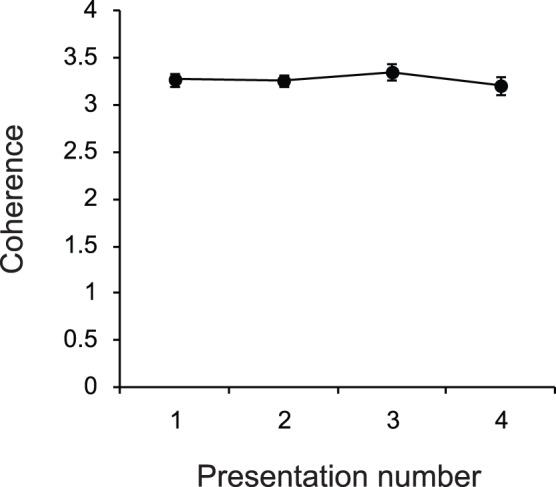
Inter-observers coherence of gaze location plotted as a function of repeated presentation of the same footage in Block 1. Error bars indicate 1 standard error of the mean (*N* = 40).

In their natural dynamic scene observation study Dorr et al. reported that inter-observer gaze coherence gradually decreased with repetitive viewing of the same video clips [24]. However we did not find this tendency in our results. Figure 6 plots the NSS value in Block 1 as a function of the number of repetitions. As shown in this figure the inter-observer coherence is constant across four-time presentations (one-way repeated measures ANOVA: *F*_3,117_ = 2.49, *p* = .06). One reason for this is that observer’s interest may be largely influenced by the context of the clips we used. That is, observing racket sports (e.g. two players are competing for a point) may have lead to high convergence of observers’ gaze onto ball events (e.g. location of hit, or bounce of the ball). Through a repetitive presentation of the same footage observers can predict the location of such events more precisely, which could result in a higher inter-observer coherence of gaze pattern. Thus, under this hypothesis, repetition of videos of the same type maintained gaze coherence because the viewing strategies that observers used converged, rather than diverged, across repetitions.

## Discussion

This study is the first of its kind to investigate the effects of goal specificity on eye-movement behavior during natural dynamic scene observation. To date, knowledge of the effects of task instruction on eye-movement behavior comes mostly from studies of eye-movement recordings taken during static scene observation.

In contrast to previous studies that have reported the effects of task goals on eye-movements [15]–[20], we did not find evidence of an influence of task instructions on eye-movement behavior. One possible reason for the discrepancy between previous findings and the present study is based on how well-defined the region of interest is. In previous studies eye-movement behaviors were compared under very different task sets [15]–[20]. For example, eye-movements were recorded for a group in which observers were asked to memorize the contents in a scene, and this was compared with a group of observers that were searching for a target item in a scene. The observers required to memorize the scene may have continued to move their eyes around the scene until the end of its presentation in order to maintain an accurate representation of as many features in the scene as possible. Those asked to search for a specific item in a scene would likely stop moving their eyes over the whole scene once they found the target. In this case the nature of the task goal is likely to have changed the region of interest in terms of ongoing processing of the scene. We speculate that, in the present study, the region of interest from moment to moment was defined by the context itself, and so this was likely to be the same for both groups while they observed the tennis clips. In other words, the key instructional difference induced by the SG task concerned increasing interest in game-point-related locations on screen, and not the intrinsically interesting locations in the presented scene. However, in every clip, it was likely that what was interesting and what was of interest converged on the same scene information for both groups because the context of the game is game-point-allocation and the events in the scene are structured around this.

On a related note, previous studies have demonstrated that visual features of motion are strong gaze attracters [8], [23], [24]. Therefore, another possible reason for the differences between previous findings and the current results is that the visual scene presented in the current experiment included a very rapidly moving object (ball) throughout the duration of each clip presentation. This clearly would have defined a region of interest in all the clips, and so it may be that the continuous movement of the ball may override the effect of goal-specificity. Note that this does not indicate that eye-movements are predominantly determined by bottom-up salient features as proposed by a theory based on visual feature saliency. In the clips we used in our study, the objects which had strong motion features were also contextually meaningful (i.e. ball and players); therefore it is difficult to separate the bottom-up factor and the top-down factor in the present stimulus set we used.

In addition to the aforementioned factors, the presence of sound may also explain why eye-movement parameters did not change despite the differences in goal specificity. Several studies have demonstrated that sound facilitates the detection of visual stimuli [38]–[42]. Among them, a study conducted by Burg et al. is most relevant to the current results [41]. They asked observers to locate a target in a jumbled, and continuously changing, visual search display. Finding targets in such complex displays is normally very difficult and time consuming. However, when the temporal changes of a target feature was synchronized with an auditory pip, then observers quickly found the target irrespective of the display size. Thus, the findings suggested that visual attention can be automatically directed to a target location via auditory cues. In the current experiment, audio footage of the tennis match was presented in synchrony with the tennis videos. Thus, the sounds of ball bounces and hits were synchronized with the visual ball events, and this might have automatically cued observer’s attention to the location of particular ball events. Therefore, in the present study the SG task could not compete with the audiovisual synchronization, which may have created powerful attention capture.

In contrast with the results of eye-tracking, the results from the subjective ranking task suggests that observers believed that they changed their attentional target as if they adopted efficient attention allocation strategies to deal with the different demands for processing information as a function of goal specificity. Although we measured the meta-attentional allocation, observers’ beliefs concerning their attention allocation strategy is concordant with previous studies which have reported that attention allocation can be adaptively changed in response to task demands [43]–[45]. The discrepancy between subjective reports and eye-movement analysis suggests that changes in meta-attentional allocation can take place without changing eye-movement behavior.

The current results suggest that changes detected while internally monitoring attention allocation do not necessarily reflect the changes that are actually occurring in eye-movement behavior. However, we take great caution in drawing this conclusion because the eye-movement parameters that were analyzed here did not directly assess where observers actually looked. We originally hypothesized that by making point-winner judgments, observers would necessarily focus more on the point-critical locations in the scene and that would result in a higher NSS value in the SG trials, which was not borne out in our results. To a first approximation the result suggests that SG did not change the locations of where observers looked. However, it might be that the frequency of looking at point-related items was actually different between SG and NSG trials (i.e. Block 1 vs. Block 2 in the test group). NSS measures how gaze locations are clustered among observers. Thus even if SG did change the most fixated locations, if the fixations in the SG trial were clustered comparably to the fixations in the NSG trial, the difference will not lead to a NSS difference between Block 1 and Block 2. A parsimonious conclusion we can derive from the NSS analysis would be therefore that goal specificity did not change the clustering of observer’s gaze locations. Nevertheless, the effect of goals on subjective reports and the lack of effect of goals on eye-movement measures indicate that changes in meta-attentional loci in response to a goal directed tasks can take place without a change to major eye-movement parameters (e.g., saccadic amplitudes, inter-saccadic intervals, and gaze coherence).

## Materials and Methods

### Ethics Statement

The experiment was approved by the local ethics committee of the University of Surrey. Written informed consent was obtained from each observer prior to the experiment.

### Observers

Forty volunteers from the University of Surrey took part in the experiment: 20 in the test group and 20 in the control group. All were naïve to the experiment’s purpose. Before the main experimental session started, observers answered a questionnaire regarding their knowledge and experiences of tennis and other racket sports (see Questionnaire S1). The answers to the pre-experimental questionnaire were used to make equivalent the observer’s knowledge and familiarity of tennis game between the two groups. They received payment and/or partial course credit for their participation.

### Stimuli

The stimuli were presented on a 19-inch CRT monitor, 60 cm in front of the observer. The clip subtended 32.7×24.8 deg in visual angle on the screen. Stimulus presentation and data acquisition were controlled by Experimental Builder (SR Research) running on a PC.

Thirty video clips were selected and cut from commercial DVDs from four major titles (1993 Wimbledon Championships ladies’ singles, 2008 Wimbledon Championships ladies’ singles, 2008 French Open women’s singles, 2008 Wimbledon Championships gentlemen’s singles). Each clip included only ‘play shots’ of the game in which a camera faced down the whole tennis court from behind the center mark. Interpolated closed shots or replays were not included in the selected clips. Camera edits (scene cuts) were not included in all of the selected clips because they could have significant effects on eye-movements [8], [23], [46]. Sound files (.wav) were also extracted from the DVDs and presented in synchronization with each clip via stereo speakers. In Block 1 ten clips were selected from the 1993 Wimbledon women’s game and presented four times in a blocked random order. The mean duration of the Block 1 clips was 8.8 sec (±1.8 SD). In Block 2**,** eight clips were selected from 1993 Wimbledon women’s final (four of which were the same clips presented in Block 1) and 12 clips were selected from three other competitions (four from each). Each of the 20 test clips was presented once in random order during the second block. The mean duration of the Block 2 clips was 9.4 sec (±1.5 SD).

### Experimental Procedure

At the start of Block 1 an instruction screen was presented. After which, in each trial, a clip was presented. When it was over a new screen appeared, in which two list boxes were presented on the left-side and the right-side of the screen. The left box included nine items, while the right box was vacant. The items listed in the left box were (1) Player A (top of the screen), (2) Player B (bottom of the screen), (3) Ball, (4) Net, (5) Horizontal line, (6) Vertical line, (7) Ball boy/girl, (8) Audience, and (9) Umpire. The initial ordering of these items was randomized for each trial. A single mouse click moved each item from the left box to the right box (or vice versa), and the moved item was placed from top to bottom in the right box. The observer’s task was to rank the items from the most attended to the least attended (higher on the list indicated more attended). After all items were ordered, observers clicked the ‘submit’ button to initiate the next trial. After all 40 trials in Block 1 were presented observers received instructions for Block 2. As with Block 1, a series of clips were presented, and after each clip the ranking screen appeared. In addition, for the test group, a check box was also presented in which observers were required to indicate which of the two players was awarded the point by clicking Player A, or Player B.

### Eye-movements Recording

Observer’s eye-movements were monitored while they were observing the tennis clips. An infrared video-based eye-tracker sampling at 1000-Hz (Eyelink 1000, SR Research) was used for eye tracking. Viewing was binocular, but only the left eye was tracked. A chin-and-forehead rest was used to stabilize observer’s head. At the start of each block calibration and validation were performed using a series of nine dots arranged in a square grid. At the start of each trial a bull’s eye was presented at the center of the screen. Observers were asked to fixate on this fixation marker and if the deviation between the measured eye position and the fixation maker was too large a recalibration was conducted.

## Supporting Information

Questionnaire S1 Questionnaire for knowledge and experiments about tennis/racket sports. Preceding the experiment we asked participants following questions to equalize their knowledge and experiments about tennis/racket sports between the test group and the control group. We assigned 1 pt for “yes” answers to each question. The average score was 4.5 (±3.4 SD) for the test group and 4.4 (±3.1 SD) for the control group, *p*>.1 by two-tailed *t*-test.
(DOC)
