# Supplementary material for: Looking to Score: The Dissociation of Goal Influence on Eye Movement and Meta-Attentional Allocation in a Complex Dynamic Natural Scene
Source: PLoS One. 2012 Jun 29;7(6):e39060. doi: 10.1371/journal.pone.0039060 (PMC3387190; doi:10.1371/journal.pone.0039060)
Supplement: Questionnaire S1 — Questionnaire for knowledge and experiments about tennis/racket sports. Preceding the experiment we asked participants following questions to equalize their knowledge and experiments about tennis/racket sports between the test group and the control group. We assigned 1 pt for “yes” answers to each question. The average score was 4.5 (±3.4 SD) for the test group and 4.4 (±3.1 SD) for the control group, p>.1 by two-tailed t-test. (DOC) [file pone.0039060.s001.doc]

**Questionnaire S1: Questionnaire for knowledge and experiments about tennis/racket sports**

Q1 “How familiar are you with the rules of tennis?”

1. I can accurately judge how an individual point has been awarded.
2. I know what the scoring system is (e.g., game, set, match)
3. I know what ‘love’ set refers to in a game of tennis
4. I know what ‘let’ refers to in a game of tennis
5. I know what ‘foot fault’ refers to in a game of tennis
6. I know what a ‘rally’ refers to in a game of tennis
7. I know what a ‘tie breaker’ refers to in a game of tennis.

Q2: Do you play/Or have you played other racket sports in the last five years (if yes, please answer the next question).

Q3: Which of the following do you / have you played in the last five years. (Please select from the options below, which are appropriate to you, you can select more than one option)

1. Squash
2. Badminton
3. Soft ball
4. Racquets
5. Matkot (Paddle ball)
6. Soft tennis
7. Tennis
